# Regulation of Autophagy in Cells Infected With Oncogenic Human Viruses and Its Impact on Cancer Development

**DOI:** 10.3389/fcell.2020.00047

**Published:** 2020-02-28

**Authors:** Tiziana Vescovo, Benedetta Pagni, Mauro Piacentini, Gian Maria Fimia, Manuela Antonioli

**Affiliations:** ^1^National Institute for Infectious Diseases “Lazzaro Spallanzani” – IRCCS, Rome, Italy; ^2^Department of Biology, University of Rome “Tor Vergata,” Rome, Italy; ^3^Department of Molecular Medicine, University of Rome “Sapienza,” Rome, Italy

**Keywords:** oncogenic (or carcinogenic) viruses, autopaghy, human papillomavirus (HPV), Merkel cell polyomavirus (MCPyV), hepatitis B and C viruses (HBV and HCV), Epstein–Barr virus (EBV), Kaposi’s sarcoma-associated herpesvirus (KSHV), human T-lymphotropic virus 1 (HTLV–1)

## Abstract

About 20% of total cancer cases are associated to infections. To date, seven human viruses have been directly linked to cancer development: high-risk human papillomaviruses (hrHPVs), Merkel cell polyomavirus (MCPyV), hepatitis B virus (HBV), hepatitis C virus (HCV), Epstein–Barr virus (EBV), Kaposi’s sarcoma-associated herpesvirus (KSHV), and human T-lymphotropic virus 1 (HTLV-1). These viruses impact on several molecular mechanisms in the host cells, often resulting in chronic inflammation, uncontrolled proliferation, and cell death inhibition, and mechanisms, which favor viral life cycle but may indirectly promote tumorigenesis. Recently, the ability of oncogenic viruses to alter autophagy, a catabolic process activated during the innate immune response to infections, is emerging as a key event for the onset of human cancers. Here, we summarize the current understanding of the molecular mechanisms by which human oncogenic viruses regulate autophagy and how this negative regulation impacts on cancer development. Finally, we highlight novel autophagy-related candidates for the treatment of virus-related cancers.

## Introduction

High-risk human papillomaviruses (hrHPV) and Merkel cell polyomavirus (MCPyV) are implicated in the development of mucosal and cutaneous cancers. Hepatitis B virus (HBV) and hepatitis C virus (HCV) are involved in approximately 80% of human hepatocellular carcinoma (HCC). Epstein–Barr virus (EBV), human herpesvirus 8 (HHV8), and human T-lymphotropic virus 1 (HTLV-1) showing lymphocytes tropism (Akram et al., 2017) are human viruses directly associated with cancer development. All these viruses encode genes essential for viral replication, which transform the host cells leading to chronic inflammation, uncontrolled proliferation, and the alteration of several cellular pathways, which are responsible for malignant transformation depending on the host genetic background and immune system response (Table 1). Autophagy is emerging as an important pathway modulated by a plethora of viruses for viral benefit (Choi et al., 2018). It is a conserved catabolic process that controls the lysosomal degradation of intracellular components. Over its role in cellular homeostasis and energy production, autophagy is a key mechanism in cellular quality control. For example, it ensures the specific removal of aggregation-prone proteins and damaged mitochondria (a process known as mitophagy), thus limiting the accumulation of abnormal aggregates and ROS production (Murrow and Debnath, 2013). Autophagy also plays an important role in innate immune defense representing a cellular response against bacterial, viral, or parasitic infections; the cellular process attempting to remove pathogens via autophagy is named xenophagy (Levine et al., 2011).

**TABLE 1:** Oncogenic viruses, viral features, oncogenes, and associated cancers.

Virus	Capsid/envelope	Genome	Viral oncogenes	Targets	Tropism	Cancer type
HPV	Icosahedral/no	Circular dsDNA	E5, E6, E7	p53, pRb	Skin, mucosa	Cervical cancer, vaginal cancer, anal cancer
MCPyV	Icosahedral/no	Circular dsDNA	T antigen (large and small)	pRb	Skin, mucosa	Merkel cell carcinoma

HBV	Icosahedral/yes	Circular dsDNA	HBx, HbsAg	p53, NF-κB, JAK/STAT	Hepatic tissue, lymphatic system	Hepatocellular carcinoma
HCV	Icosahedral/yes	ssRNA	Core protein, NS3/4, NS5B, NS5A	NF-κB, p53	Hepatic tissue, lymphatic system	Hepatocellular carcinoma

KSHV	Icosahedral/yes	Linear dsDNA	vFLIP, vCyclin D LANA, Kaposin B/v-IL6, vGPCR/vBcl2, K7	p53, pRb, GSK-3β	B lymphocytes, lymphatic epithelia	Kaposi’s sarcoma, primary effusion lymphoma
EBV	Icosahedral/yes	dsDNA	LMP-1, LMP-2, BHRF1/BALF1, EBNA-3C, Rta	NF-κB, JAK/STAT, c-myc	B lymphocytes, lymphatic epithelia	Burkitt’s lymphoma, nasopharyngeal cancer, Hodgkin and non-Hodgkin’s lymphoma
HTLV-1	Icosahedral/yes	ssRNA	Tax, HBz	NF-κB	CD4^+^ T-lymphocytes, lymphatic epithelia	Adult T-cell lymphoma

*HPV, human papilloma virus; HCV, hepatitis C virus; HBV, hepatitis B virus; EBV, Epstein–Barr virus; MCV, Merkel cell polyomavirus; KSHV, Karposi’s sarcoma herpesvirus; HTLV-1, human T-lymphotrophic virus.*

**FIGURE 1:**
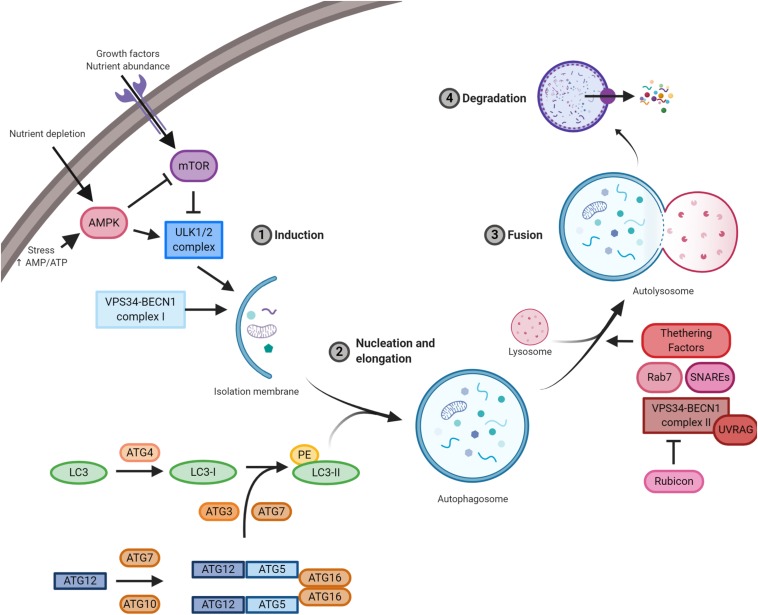
A schematic illustration of the autophagic pathway. Nutrient-rich conditions stimulate mTOR kinase activation and its inhibition of the ULK1/2 complex. Low nutrients and/or growth factors stimulate AMPK, promote mTOR inhibition, and allow ULK1/2 complex to stimulate autophagy. ULK1/2 and VPS34/BECLIN 1 complexes induce autophagy and the formation of the isolation membrane. Then, autophagy-related gene (ATG)5/12 complex and LC3 promote autophagosome formation. Subsequently, autophagosomes fused with lysosomes and the contained material is finally degraded by lysosomal proteases. Essential complexes in the formation of autolysosomes are soluble N-ethylmaleimide-sensitive factor attachment protein receptors (SNAREs), Rab7, tethering factors, and VPS34/BECLIN 1 complex II (containing UVRAG) and its negative regulator Rubicon.

Molecular mechanisms orchestrating autophagy were initially described in *Saccharomyces cerevisiae*. To date, more than 30 autophagy-related genes (ATGs) have been characterized (Figure 1; Amaravadi et al., 2016). The upstream cellular sensor, which mediates autophagy activation, is the kinase mTOR; it perceives signals coming from the external environment through the membrane receptors, as well as from the cellular context (Yin et al., 2016). Growth factors, besides nutrient-rich conditions, promote mTOR activity, which, in turn, stimulates cell growth and blocks autophagy by negatively phosphorylating the downstream effectors of autophagy, i.e. components of ULK1 and BECLIN 1 complexes (Menon and Dhamija, 2018). mTOR inhibition is the canonical stimulus of autophagy initiation and results in the downstream activation of the kinase ULK1. Active ULK1 positively regulates BECLIN 1 complex I by phosphorylation, resulting in the production of phosphatidylinositol 3-phosphate (PI3P) by the kinase VPS34 on nascent autophagosomes (Menon and Dhamija, 2018). Both ULK1 and BECLIN 1 complexes are positively regulated by the cofactor AMBRA1, which is required for their regulative ubiquitination (Nazio et al., 2013; Xia et al., 2014; Di Rienzo et al., 2019). Moreover, AMBRA1 regulates the temporal dynamics of autophagy response being ubiquitinated by Cullin 4 and degraded by the proteasome, thus avoiding excessive self-digestion (Antonioli et al., 2014). The isolation membrane of forming autophagosomes expands, engulfs intracellular components, and finally closes. During the nucleation and the elongation of autophagic vesicles, the crucial protein LC3 is loaded on both luminal and cytoplasmic membranes of the autophagosomes (Brier et al., 2019). This event needs an E1–E3 ubiquitination-like reaction cascade, where ATG4 forms LC3-I by cleavage, which is subsequently lipidated by the addition of phosphatidylethanolamine (PE) through the coordinated action of ATG3, ATG7, and ATG5-12 proteins (Geng and Klionsky, 2008). Once formed, the autophagosomes move along microtubules through the dynein–dynactin complex to the perinuclear region, where they fuse with lysosomes leading to the formation of autolysosomes; at this point, the contained material is degraded and made again available to cell metabolism (Zhao and Zhang, 2019). The fusion step is a thinly regulated process, and its functionality is essential for the fulfillment of autophagy; three sets of protein families – Rab GTPases (e.g. Rab7), membrane-tethering complexes, and soluble N-ethylmaleimide-sensitive factor attachment protein receptors (SNAREs) – cooperate with BECLIN 1 complex II (e.g. UVRAG) to complete the autophagy process (Nakamura and Yoshimori, 2017).

### Autophagy in Viral Infection and Cancer Development

During viral infections, autophagy is a crucial cellular response, which represents an innate defense against pathogens, attempting to ensure virus depletion and cell survival (Jackson, 2015). The continuous host–pathogen co-evolution leads autophagy to assume double-faceted aspects with both anti-viral and pro-viral properties depending on the virus, cell type, and cellular environment. On one hand, autophagy directly targets viruses for degradation, also promoting inflammation, cytokine response, antigen presentation for pathogen recognition, and removal (Levine et al., 2011). On the other hand, some viruses hijack autophagic machinery ensuring their survival, replication, and propagation (Ahmad et al., 2018). Indeed, the activation of autophagy could be beneficial for other viruses; for example, it has been observed that specific proteins of poliovirus (i.e. 2BC and 3A), HBV (i.e. X protein), HCV (i.e. NS4B, NS5A, and NS5B), EBV (i.e. Rta), and SV40 (i.e. T antigen) stimulate autophagy induction (Jackson, 2015). However, the inhibition of autolysosome fusion is one of the most frequent mechanisms by which viruses usurp autophagy, thus ensuring a remarkable pool of membranes for viral particle assembly (de Armas-Rillo et al., 2016). In addition, the fate of autophagosomes could be redirected to the plasma membrane for the release of viral particles, further ensuring pathogen propagation (Choi et al., 2018).

**FIGURE 2:**
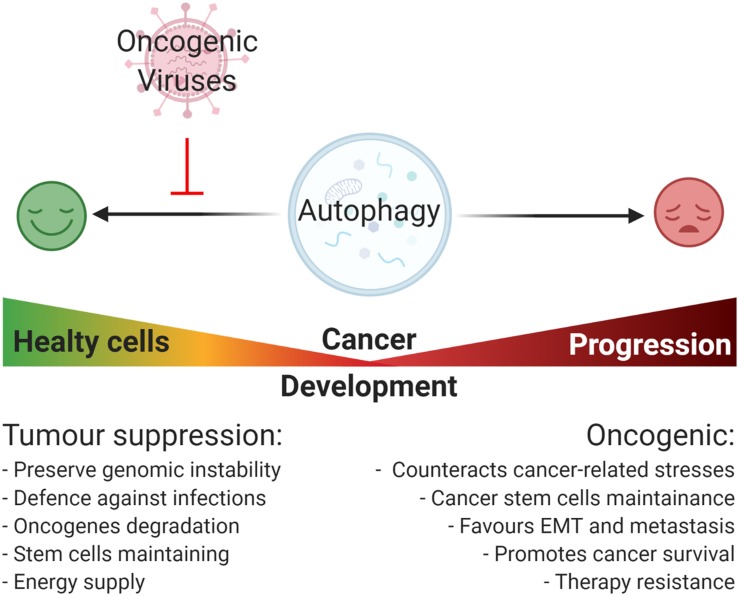
Oncogenic viruses and their general impact on autophagy-regulated tumorigenesis. Autophagy plays a Janus-faced role on cancer development: on one hand, it acts as a tumor suppressive mechanism by preserving genomic instability, promoting the degradation of damaged organelle and promoting the degradation of cellular oncogenes, and the removal of pathogens; on the other hand, once cancer is established, autophagy acts as an oncogenic mechanism promoting cancer progression through the reduction of cancer-related stresses, the maintenance of cancer cells stemness potential, and promoting ETM transition, metastasis, and resistance to therapy. In this context, most of the oncogenic viruses possess the capability to inhibit autophagy mainly promoting viral replication and cellular maintenance, though this favors cancer onset.

Similar to viral infections, autophagy plays a dual role in cancer acting as a tumor suppressor, as well as a tumor-promoting mechanism during malignant progression and chemoresistance (Yun and Lee, 2018). Defective autophagy causes diminished cell quality control associated to genomic damage, metabolic stress, and tumorigenesis (Lorin et al., 2013). By contrast, the upregulation of autophagy is beneficial for malignancy progression helping cancer cells to get over metabolic stresses and cytotoxicity of chemotherapy (Kögel, 2012). Importantly, autophagy also contributes to stemness maintenance of cancer stem cells, playing a role in tumor recurrence and resistance to therapy (Nazio et al., 2019; Figure 2). This review is focused on how the regulation of autophagy by oncogenic viruses contributes to tumorigenesis. A higher emphasis is posed on those mechanisms involved in host – pathogen interaction that could inform novel therapeutic strategies based on autophagy modulation to counteract virus-associated cancers.

## Cutaneous and Mucosal Oncogenic Viruses

### Human Papilloma Viruses (HPVs)

Human papilloma viruses belong to the *Papillomaviridae* family, which includes 16 different genera of circular small dsDNA, non-enveloped viruses that have a tropism for the mucosal or cutaneous epithelia. To date, around 200 human papillomaviruses have been identified, classified into two different sub-groups depending on the epidemiological features, i.e. high-risk (HR) and low-risk (LR) HPVs; in particular, 12 mucosal HPV types (HPV16, 18, 31, 33, 35, 39, 45, 51, 52, 56, 58, and 59) belong to HR-HPVs and have been described as etiological agents for the development of human malignancies (IARC Group 1), affecting several anatomical districts such us cervix, vagina, vulva, anus, penis, head–neck, and oropharynx and causing squamous epithelial cancers (Gheit, 2019). Similar to HBV and MCPyV, HPV is involved in carcinogenesis in a state called “pseudo-latency,” where the viral genome is integrated in the host cells, produces oncoviral proteins, and drives mutagenesis, although unable to replicate (Chang et al., 2017). Three major early genes are involved in HPV-induced malignancies, i.e. E5, E6, and E7 (Table 2).

**TABLE 2:** Oncogenic viruses, viral genes, and their impacts on autophagy and cancer regulation.

Virus	Gene	Autophagy	Effects on autophagy	Tumorigenesis	Host targets	References
HPV	E5	▼ Induction, nucleation and elongation	▼ BECLIN 1, ATG5, LC3, ULK2, ATG4a, and ATG7	▲ Cell proliferation ▼ Apoptosis	▲ EGFR ▼ TNFL and CD95L	Belleudi et al., 2015; de Freitas et al., 2017
	E6/E7	▼ Autolysosome formation	▲ LC3II, p62	▼ Apoptosis ▲ Cell proliferation	▼ p53 ▲ pRb	Zhou and Münger, 2009; Mattoscio et al., 2017
MCPyV	st-ag/LT-ag	▼ Induction, nucleation, and elongation	▼ ATG7, BECLIN 1, and p62	▼ Apoptosis ▲ Cell proliferation	▼ p53 ▼ pRb	Kumar et al., 2019
HCV	Core protein	▼ Mitophagy ▲ Induction	▼ Parkin translocation to mitochondria ▲ LC3, ATG12	▲ Cell proliferation ▲ Angiogenesis	▲ NF-κB ▲ HIF1α, VEGF, MMPs	Abe et al., 2012; Vrancken et al., 2012; Akram et al., 2017
	NS3/4	▲ Induction	Interaction with IRGM	▼ Genetic stability ▼ Apoptosis	▼ DNA repair ▼ p53 pathway	Lai et al., 2008; Vescovo et al., 2016
	NS5B	Not identified	Interaction with ATG5	▲ Cell cycle progression	▼ Rb	Munakata et al., 2005
	NS5A	▲ Induction ▲ Mitophagy	▲ BECLIN1 ▲ Parkin translocation to mitochondria	▼ Apoptosis ▼ Genetic stability	▼ p53, Bid, CASP3 activation ▲ ROS	Ghosh et al., 2000; Lan et al., 2002; Simonin et al., 2009; Vescovo et al., 2016
HBV	HBx	▲ Induction	▲ BECLIN 1, VPS34	▲ Proinflammatory cytokines ▲ Tumor invasion and angiogenesis ▲ Cell proliferation	▲ NF-κB VEGF, MMPs ▲ JAK-STAT signaling	Liu et al., 2010; Luo et al., 2015; Akram et al., 2017
		▼ Autolysosome maturation	▼ Rab7	▼ Apoptosis ▲ Cell proliferation	▼ p53 ▼ pRb	Zemel et al., 2011
		▲ Mitophagy	▲ LONP1 Parkin translocation to mitochondria	▼ Genetic stability ▲ Tumor cell growth	▼ DNA Repair ▲ ROS ▲ Wnt signaling	Cheng et al., 2010; Jung and Kim, 2013; Geng et al., 2015
KSHV	vFLIP	▼ Nucleation and elongation	▼ ATG3	▼ Apoptosis	▼ CASP8 ▼ NF-kB	Ganem, 2007; Lee et al., 2009
	vCyclinD	▲ Induction	▲ AMPK	▼ Cell cycle deregulation	▲ v-Cyclin-CDK6	Leidal et al., 2012
	LANA	▼ Induction	▼ p53	▲ Cell proliferation ▼ Apoptosis	▼ pRb ▼ p53	Friborg et al., 1999; Ganem, 2007
	Kaposin B/v-IL6	▼ Induction	▲ STAT3	▲ KSHV lytic cycle activation	▲ p53/p21	Santarelli et al., 2014, 2019; King, 2013
	vGPCR/vBcl2	▼ Induction	▲ PI3K/AKT/mTOR ▼ BECLIN 1 and ATG14L	▲ Apoptosis	Viral homolog of cellular Bcl-2	Pattingre et al., 2005; Bhatt and Damania, 2012; Zhang et al., 2015
	K7	▼ Autophagosome maturation	▲ Rubicon/BECLIN 1	▼ Apoptosis	K7 acts like IAP	Moore, 2007; Liang et al., 2013
EBV	LMP-1	Not identified	Autophagic substrate	▲ Cell proliferation ▼ Apoptosis	Mimics a constitutively active CD40R	Mosialos et al., 1995; Lee and Sugden, 2008
	LMP-2	▼ Induction ▲ Autophagosomes formation	▲ PI3K/AKT/mTOR ▲ ATG5/12, LC3	▲ Cell proliferation ▼ Apoptosis	▲ MYC ▼ p27^Kip^	Fish et al., 2014; Cen and Longnecker, 2015; Fotheringham and Raab-Traub, 2015; Luo and Ou, 2015
	BHRF1/BALF1	▼ Induction	▼ BECLIN 1	▼ Apoptosis	▲ Viral homolog of cellular Bcl-2	Farrell, 2019
	EBNA-3C	▲ Autophagosome formation	▲ ATG3, 5, 7	▲ Cell proliferation ▼ Apoptosis ▼ Apoptosis	▼ BIM, p16	Akram et al., 2017; Bhattacharjee et al., 2018
	Rta	▲ Autophagosome formation	▲ ERK1/2	▲ Cell survival	▲ ERK1/2	Balmanno and Cook, 2009; Hung et al., 2014
	Unknown	▼ Autophagosome maturation				Granato et al., 2014
HTLV-1	Tax-1	▲ Autophagosome formation ▼ Autophagosome maturation	▲ BECLIN 1 ▲ NF-kB	▼ Apoptosis ▲ Cell proliferation	▲ CREB	Yin et al., 1995; Tang et al., 2013; Ren et al., 2015
	HBz	▼ Induction	▲ mTOR ▼ GADD34	▼ Apoptosis ▲ Cell proliferation	▼ BIM ▲ BDNF, TRkB	Mukai and Ohshima, 2014; Polakowski et al., 2014; Tanaka-Nakanishi et al., 2014

The oncogenic activity of E5 includes its capability to stimulate EGF-mediated cell proliferation and, on the other hand, to affect apoptotic cell death induced by both tumor necrosis factor ligand (TNFL) and CD95 ligand (CD95L) (Kabsch and Alonso, 2002). E5 acts as viroporin regulating cellular ion homeostasis and vesicle trafficking, resulting in the alteration of immunoresponse mechanisms, such us inflammation and antigen presentation (DiMaio and Petti, 2013; de Freitas et al., 2017). A central role in the HPV-linked carcinogenesis is played by E6 and E7 oncoproteins, which inhibit p53 and pRb (retinoblastoma), respectively, affecting apoptosis inhibition and promoting proliferation (Hoppe-Seyler et al., 2018). To date, it has been extensively shown that E6 and E7 cooperate in cancer development, especially in case the expression of the viral inhibitor E2 is lost (Hoppe-Seyler et al., 2018). It has been shown that E6 alters the conformation of the E3-ubiquitin ligase E6AP (E6-associated protein), which, associated to E6, allows the ubiquitination and the proteasomal degradation of the oncosuppressor p53 (Scheffner et al., 1990; Martinez-Zapien et al., 2016). Similarly, E7 mediates the degradation of Rb through its cleavage by calpains and the subsequent proteasomal degradation mediated by Cullin 2, an E3-ubiquitin ligase responsible for the ubiquitination of E7 targets (Darnell et al., 2007; White et al., 2012b). The downregulation of Rb directly affect the cell cycle through the release of the transcriptional factor E2F, thus promoting cancer development (Hoppe-Seyler et al., 2018). In addition, several cellular targets of E6 and E7 have been extensively analyzed by mass spectrometry, highlighting additional host-cellular protein-protein interactions that could be relevant in HPV-related cancer pathogenesis (Neveu et al., 2012; White et al., 2012a, b).

**FIGURE 3:**
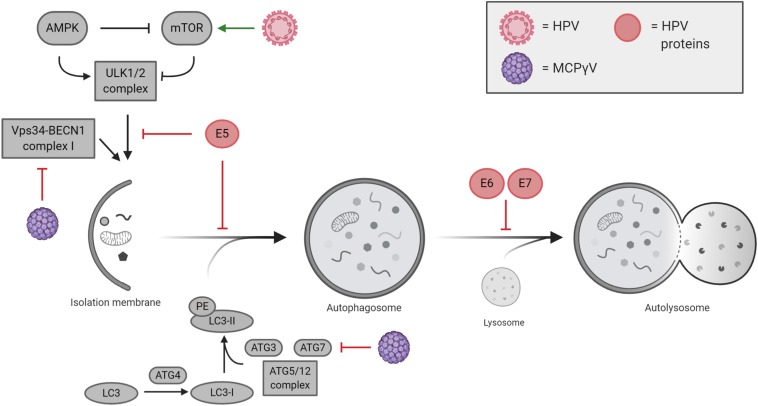
Cutaneous and mucosal oncogenic viruses and their impacts on autophagy regulation. Human papillomavirus (HPV)-mediated modulation of autophagy is mostly performed by viral oncogenes E5, E6, and E7, which act at different levels during autophagy execution. Merkel cell polyomavirus (MCPyV) negatively regulates autophagy by inhibiting VPS34/BECLIN 1 complex I and ATG7.

Although many of the cellular mechanisms behind HPV-related tumorigenesis have been extensively characterized, some aspects still need further investigation including those responsible for autophagy regulation. It is characterized that HPV mainly blocks autophagy induction to avoid viral degradation during *de novo* infection (Mattoscio et al., 2018); however, only a small number of studies correlate the reduction of autophagy with cancer development in HPV pseudo-latency infections. Indeed, HPV directly impacts the autophagic machinery in the host cells, thus ensuring viral replication and life cycle (Figure 3). The manipulation of autophagy by HPV occurs immediately following the initial binding of viral L1 and L2 proteins to heparan sulfate proteoglycans (HSPGs), present on the cell surface, targeting the epidermal growth factor receptor (EGFR) (Mattoscio et al., 2018). Activated EGFR promotes the phosphorylation of both PTEN and AKT, which in turn positively regulate the activity of the mTOR complex 1, a main inhibitor of autophagy (Surviladze et al., 2013). The ability of HPV to rapidly inhibit autophagy represents a key element for viral internalization, which occurs by clathrin-dependent endocytosis (Cossart and Helenius, 2014). Once internalized, HPV can be degraded by late endosomes (Sapp and Bienkowska-Haba, 2009), which can merge with autophagosomes to form amphisomes and eventually fuse to lysosomes for the degradation of engulfed materials (Nakamura and Yoshimori, 2017), thus pointing out a relevant aspect in HPV survival by blocking the degradation of viral particles via autophagy.

The ability of HPV in modulating autophagy response has been well characterized during *de novo* infection (Mattoscio et al., 2018); nevertheless, less is known in pseudo-latency and cancer development, opening questions about the HPV-mediated inhibition of autophagy during the transformation of the mucosal epithelia. It is now emerging that HPV16 E5 expression in keratinocytes reduces the formation of autophagosomes upon serum starvation and keratinocyte growth factor (KGF) by functionally inhibiting the activity of p53, which in turn affects the transcription of several autophagy-related genes involved in autophagosome formation (*i.e. BECLIN 1, ATG5, LC3, ULK2, ATG4a, and ATG7*) (Belleudi et al., 2015). Differently, HPV16 E6/E7 expression in keratinocytes was described to impair the formation of autolysosomes causing the accumulation of lipidated LC3 containing autophagosomes and of the autophagic substrate, p62 (Zhou and Münger, 2009; Mattoscio et al., 2017). Interestingly, Mattoscio et al. show an accumulation of p62 in the skin of high-grade squamous intraepithelial lesion (HSIL), where HPV infection causes pre-cancerous injury (Mattoscio et al., 2017), highlighting the concept that although the expression of HPV oncogenes is causative in the development of HPV-related tumors, it is not sufficient for carcinogenesis. To this regard, it is well characterized that genomic instabilities and/or failure of the immune system concourse in HPV-related cancer development (Mesri et al., 2014). In cervical cancer, the role of autophagy in HPV-mediated tumors was described *in vivo* in cervical keratinocytes, where the depletion of HPV E6/E7 associates with an upregulation of autophagy genes (Hanning et al., 2013). Furthermore, the comparison between HPV16 transgenic mice (K14E6/E7) and non-transgenic mice (FVB/N) display an increased susceptibility to chemical carcinogen, 7,12-dimethylbenz[a]anthracene (DMBA) in HPV16 animals, which correlate with an inhibition of autophagosome maturation (Carchman et al., 2016). In line with this, the occurrence of anal tumors induced by DMBA in mice is increased both by the chemical and genetic inhibition of autophagy (Rademacher et al., 2017, 2018). To this regard, Carchman et al. (2016) argue that autophagy impairment is mainly associated in early anal cancer development caused by HPV (low-grade dysplasia). On the other hand, autophagy appears restored and higher in late-stage cancers (high-grade dysplasia and invasive carcinoma), opening to the possibility that, as demonstrated for different cancer types, autophagy activation supplies stressful conditions in cancer microenvironment (e.g. hypoxia, inflammation, and cytokines) and promotes cancer progression by counteracting cellular stress (Yun and Lee, 2018). In oropharyngeal squamous cell carcinoma (OPSCC), it has been recently shown that HPV-negative OPSCC patients display higher levels of the autophagic marker LC3B, analyzed by immunohistochemistry; vice versa HPV-positive tumors correlate with a reduction in LC3B protein levels suggesting that autophagy is affected in HPV-positive cancers (Lai et al., 2018). Lai et al. (2018) correlated the overall survival of HPV-positive and -negative OPSCC patients with the expression of LC3B indicating that high autophagy is with the reduced survival observed in HPV-negative OPSCC patients. It has been demonstrated that the inhibition of autophagy by Chloroquine treatment, as well as by RNAi, enhances the response to both radio- and chemotherapy in several cancer cell lines, such us breast carcinoma, colorectal cancer, non-small cell lung cancer, and glioma stem cells (Apel et al., 2008; Lomonaco et al., 2009; He et al., 2014; Schonewolf et al., 2014). In line with these observations, a recent study has reported the development of polymeric nanomicelles to simultaneously delivery the anticancer drug doxorubicin (DOX) and the autophagy inhibitor, LY294002, in oral cavity (tongue) squamous cell carcinoma (SCC) cells, opening new perspectives in the therapy of these cancers by directly affecting autophagy (Saiyin et al., 2014).

### Human Polyomaviruses and Merkel Cell Polyomavirus (MCPyV)

To date, 13 human polyomaviruses (HPyV) were identified (i.e. BKPyV, JCPyV, KIPyV, WUPyV, MCPy, HPyV6, HPyV7, TSPyV, HPyV9, HPyV10, STLPyV, HPyV12, and NJPyV-2013). These are non-enveloped viruses with a circular dsDNA encoding regulatory and structural proteins (Moens et al., 2015). Among them, BKPyV, JCPyV, and MCPyV possess oncogenic properties (Moens et al., 2007; Borchert et al., 2014; Spurgeon et al., 2015; Verhaegen et al., 2015). However, only MCPyV has been directly associated with cancer development with 80% of the human Merkel cell carcinoma (MCC) tumors positive with the MCPyV genome (Spurgeon et al., 2015). MCC is an uncommon skin cancer, typically affecting immunosuppressed individuals and people >65 years. MCC is an aggressive cancer with a poor prognosis, the 5-year survival is 51% for the local disease and 14% for the distant disease (Coggshall et al., 2018). The link of MCPyV with cancer development resides in the expression of two viral proteins (Table 2), large T-antigen (LT-ag) and small t-antigen (st-ag), which are able to inhibit the tumor suppressors pRb and p53, and the protein phosphatase 2A (PP2A), respectively (Moens et al., 2015). In addition, it has been extensively described that MCPyV evades the innate immune system inhibiting the expression of the transcription factor C/EBPβ, which in turn downregulates the expression of Toll-like receptor 9 (TRL9) and cytokines (e.g. IL-6, IL-8, and TNF-α) (Spurgeon et al., 2015). Moreover, MCPyV-positive MCCs also show a strong reduction in the cellular surface exposition of MHC-I (Paulson et al., 2014), which parallels to a downregulation of natural killer (NK)-activating receptors (i.e. NKG2D) (Chretien et al., 2014) ensuring immune evasion and avoiding NK targeting. Importantly, also NF-kB-mediated transcription is affected by st-ag of MCPyV, due to the interaction of st-ag with NEMO, PP2A, and PP4C, which prevents the nuclear translocation of NF-kB (Griffiths et al., 2013).

In the context of MCPyV infection, autophagy is emerging as a mechanism regulated by the virus and important in cancer development (Figure 3). MCPyV reduces the expression of several genes involved in autophagy induction and in the formation of autophagosomes. By contrast, as response to human polyomaviruses, it has been described that autophagy specifically sustains active BKPyV infections, a mechanism that could be likely extended to other viruses (Bouley et al., 2014). However, focusing on oncogenic polyomaviruses, it has been recently published that T-antigens of MCPyV promote the expression of cellular microRNAs (i.e. miR-375, miR-30a-3p, and miR-30a-5p) in MCC cell lines, which in turn target the expression of autophagy-related genes such us *ATG7*, *BECLIN 1*, and *p62* (Kumar et al., 2019). In addition, Kumar et al. show that MCPyV-positive MCC cancers display reduced levels of both ATG7 and p62 highlighting a new prospective in understanding how MCPyV promotes tumorigenesis. In line with this, the MCPyV microRNA, MCV-miR-M1-5p, was predicted to target human AMBRA1 gene (Lee et al., 2011), an important factor involved both in autophagy and cell death (Fimia et al., 2013; Antonioli et al., 2017). Of relevance, it is well characterized that st-ag of several HPyV (MCPyV included) interacts with the protein phosphatase PP2A (Kwun et al., 2015; Moens et al., 2015), a tumor suppressor involved in the regulation of c-Myc stability, which links autophagy to cell proliferation and tumorigenesis via AMBRA1 (Cianfanelli et al., 2015). Altogether, the data reported in the literature highlight that, although MCPyV infection could be initially sustained by active autophagy, its inhibition could represent one of the mechanisms leading to MCC tumor development. However, further studies are needed to support these preliminary evidences.

## Hepatotropic Oncogenic Viruses

**FIGURE 4:**
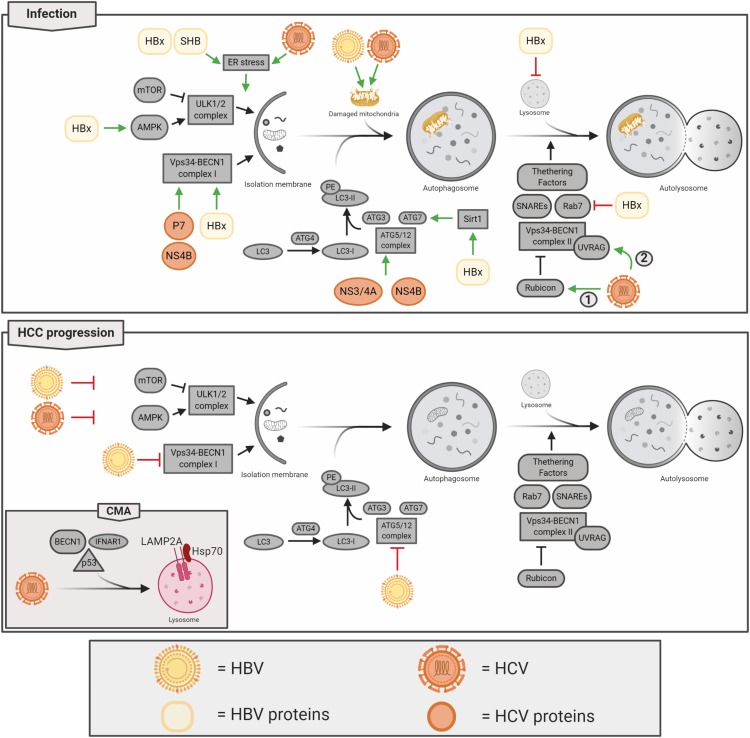
Hepatotropic oncogenic viruses and their impact on autophagy regulation. Hepatitis B virus (HBV) and hepatitis C virus (HCV) interfere with the autophagy pathway at different steps and depending on the stage of their disease. During infection, both viruses induce autophagosome formation and, at the same time, impair the maturation of the autophagic vesicles to favor viral replication. In particular, at the early stage of infection, HCV is able to block autophagy through Rubicon upregulation (1), while later, the virus restored autophagy completion through UVRAG induction (2). HBV and HCV induce mitophagy. In hepatocellular carcinoma (HCC) development and progression, autophagy is mostly inhibited by both viruses, but HCV mediates the activation of chaperone-mediated autophagy (CMA).

Hepatitis B virus and HCV are hepatotropic viruses, whose infections represent a major global public health problem, as according to the World Health Organization (WHO), approximately 257 million and 71 million people, respectively, are chronically infected worldwide (Global Hepatitis Report 2017, WHO). HBV and HCV are considered the leading causes of liver cancer being responsible for 80% of hepatocellular carcinoma (HCC) (Zamor et al., 2017). In HBV and HCV chronic-infected people, the viruses can promote the development of liver fibrosis, cirrhosis, and HCC. HCC progression consists in a complex multifactorial process involving genomic instability, immune response, and direct effect of viral proteins, which may interfere with a wide range of intracellular pathways involved in carcinogenesis, such as autophagy (Tarocchi et al., 2014; Vescovo et al., 2016). It is well established that, in order to favor their replication, HBV and HCV can positively regulate the autophagy process, both directly, through the influence of viral elements on autophagic proteins, and indirectly, through the activation of cellular stress responses, which, in turn, stimulate autophagy. Notably, in HBV- or HCV-infected cells, upregulated autophagy, on one hand, acts to mitigate cellular stresses induced by viruses, in order to promote cell survival and, on the other hand, contribute to modulate host immune response to trigger inflammation or favor viral immune evasion (Khan et al., 2018). In addition, HBV and HCV cause disruption of mitochondrial dynamics and promote mitophagy, which regulates mitochondrial homeostasis and results in a reduction of apoptosis in infected cells. Considering the central role of mitochondria in innate immune signaling, it is possible to speculate that through mitophagy, viruses may affect innate immunity (Kim et al., 2018). Hence, in promoting cell survival and interfering with host immune response, bulk as well as selective autophagy play a crucial role in virus persistence; thus, both mechanisms are strictly involved in chronic infection establishment and virus-mediated pathogenesis progression in the liver (Vescovo et al., 2014; Khan et al., 2018; Xie et al., 2018). Although many evidences have demonstrated that autophagy supports early phases of HBV and HCV infection (Tanida et al., 2009; Zhang et al., 2014), the inhibition of autophagic pathway has been observed in HBV or HCV-related cancer, suggesting that an impairment in autophagy could contribute to HCC onset (Figure 4; Kotsafti et al., 2012; Saito et al., 2016). However, the role of autophagy in the development and progression of HBV or HCV-mediated HCC is still widely debated, and further studies are needed to clarify this topic. In the next sections, we describe in detail the interplay between autophagy, HBV or HCV infection, and HCC.

### Hepatitis B Virus

Hepatitis B virus is an enveloped virus with partially doubled stranded and relaxed circular DNA (rcDNA), belonging to the *Hepadnaviridae* family. During infection, rcDNA is converted to covalently close circular DNA (cccDNA), which stably remains in the nucleus. From cccDNA, viral pregenomic RNA (pgRNA) is transcribed and enclosed into viral capsids with the HBV polymerase. Reverse transcription of the pgRNA takes place within the nucleocapsid, resulting in rcDNA or double-stranded linear DNA (dslDNA) forms. These nucleocapsids are either enveloped and secreted as virions or transported to the nucleus to add or replenish intranuclear cccDNA pool (Seeger and Mason, 2015). Intranuclear dslDNA HBV genomes could integrate into the host cell genome, and this event is crucial for pathogenesis and HCC development. cccDNA also serves as template for the transcription of all viral mRNAs, which encodes seven viral proteins including core (HbcAg), hepatitis B envelope antigen (HBeAg), viral polymerase, large (LHBs), medium (MHBs), and small (SHBs) variants of the surface antigens (HBsAg), and regulatory X protein (HBx) (Seeger and Mason, 2015). Mainly HBx and SHB proteins are involved in autophagy activation, which is exploited by HBV to positively regulate its DNA replication, both *in vitro* and *in vivo* (Sir et al., 2010b; Li et al., 2011; Tian et al., 2011; Zhong et al., 2017; Fu et al., 2018).

The envelope protein SHB translocates into the endoplasmic reticulum (ER), where, it is responsible for a massive ER stress induction (Li et al., 2011). To attenuate ER stress, infected cells activate the unfolded protein response (UPR), which is orchestrated by three signaling pathways initiated by the stress sensors inositol-required enzyme 1 (IRE1), protein kinase RNA-like ER kinase (PERK), and activating transcription factor 6 (ATF6) (Li et al., 2011). All these mechanisms are able to trigger autophagy, and interestingly, the inhibition of any of them constrains LC3 lipidation in HBV-expressing cells (Li et al., 2011). Moreover UPR-induced autophagy is crucial for the envelopment of HBV nucleocapsids. In addition to SHB protein, ER stress and UPR could also be stimulated by HBx protein (Li et al., 2007).

HBx is a multifunctional oncoprotein involved in viral pathogenesis and carcinogenesis. The oncogenic properties of HBx are related to its ability to subvert many cellular pathways such as transcription, proliferation, signal transduction, genetic stability, apoptosis, p53, and pRb suppression and autophagy, with both early and late stages of the process being strongly influenced (Geng et al., 2015; Table 2). In detail, HBx promotes autophagosome formation, upregulating BECLIN 1 expression, as well as BECLIN 1 phosphorylation by increasing the activity of death-associated protein kinase (DAPK) (Tang et al., 2009; Zhang et al., 2014). High levels of BECLIN 1 stimulate VPS34 to produce PI3P, which is required for autophagosomal membrane biogenesis (Tang et al., 2009). Furthermore, HBx is also able to activate the kinase activity of VPS34 by direct protein–protein interaction, independent of BECLIN 1 levels (Sir et al., 2010a). In addition to BECLIN 1/VPS34, HBx has a strong influence on other cellular signaling pathways involved in autophagy induction, such as MAPK, ROS/JNK, and AMPK pathways. HBx has been reported to trigger all the three pathways of MAPK, i.e. ERK, p38 MAPK, and JNK (Nijhara et al., 2001). In particular, the inhibition of JNK signaling impairs the HBx-mediated phosphorylation of Bcl2, and consequently, the interaction of Bcl2 with BECLIN 1 persists, thus avoiding autophagy induction (Zhong et al., 2017). Moreover, the HBx-mediated ROS production may stimulate the autophagic process via AMPK or ROS/JNK (Zhong et al., 2017). In this context, HBx can interact with Sirt1, modulating the acetylation status of key proteins required for autophagy in ROS/JNK pathway. The activity of Sirt1 is impaired by mir-141, which is responsible for the suppression of autophagy and HBV replication (Ou et al., 2014; Deng et al., 2017).

Despite the strong induction of the autophagic process mediated by HBx, it does not correlate to an increased rate of lysosomal degradation of autophagic substrates, indicating that autophagy is impaired during the late stages. Indeed, HBx is able to repress the activity of the lysosomal proton pump V-ATPase by interacting with its VD1 subunit, thus impairing the acidification of lysosomes and then autolysosome degradation (Liu et al., 2014). HBV is also able to interfere with autophagic degradation by altering the expression of Rab7, a protein crucial for lysosomal maturation (Zhou et al., 2016). In this contest, the accumulation of potentially harmful autophagic substrates may be crucial for the onset of HBV-induced HCC. Although these data highlight the ability of HBV to induce an incomplete autophagy process, Kim and colleagues have demonstrated that mitophagy is activated in cells expressing full-length HBV genome or HBx alone, suggesting that bulk and selective autophagy are differentially regulated by the virus (Kim et al., 2013b). In detail, the authors have found that HBV simulates mitochondrial fission through the phosphorylation and activation of DNML1/Dpr1 protein. In addition, they showed that HBV upregulates the expression of two essential effectors of mitophagy, Parkin and PINK, and that HBV or HBx alone are able to induce Parkin translocation to damaged mitochondria, which represents the initiation step of mitophagy (Kim et al., 2013b). As the maintenance of cellular homeostasis by targeting damaged mitochondria for autophagic degradation acts to attenuate virus-induced apoptosis, mitophagy promotes cell survival and consequently virus persistence. Furthermore, HBx is also able to induce Parkin-mediated mitophagy through modulating Lon Peptidase 1 (LONP1) in malignant liver cells during starvation. Since nutrient deprivation is a common phenomenon occurring in tumor microenvironment, mitophagy could act as a pro-tumoral mechanism, resulting in reduced apoptosis of transformed cells (Huang et al., 2018). Notably, HBV-induced Parkin activation attenuates innate immunity, as Parkin, interacting with mitochondrial antiviral-signaling protein (MAVS), mediates MAVS signalosome impairment, which results in blocked IFN production (Khan et al., 2016).

Another molecular mechanism, which interferes with innate immunity and is important for HBV persistence, is performed by HBx protein. In detail, HBx, acting as an autophagic adaptor, promotes the degradation of death receptor tumor necrosis factor receptor superfamily member 10B (TNFRSF10B) by the autophagic machinery, thus evading the recognition of infected cells by immune cells (Shin et al., 2016).

Persistent HBV infection establishes chronic liver inflammation, which can be directly sustained also by autophagy. Indeed, HBx-induced autophagy plays a crucial role in increasing NF-κB-mediated production of pro-inflammatory cytokines, such as IL8, CXCL2 and in particular IL6 (Luo et al., 2015), which, at high levels, is predictive of HCC progression in patients with chronic hepatitis (Wong et al., 2009).

In contrast to the high induction of autophagy during HBV infection, it has been demonstrated that autophagy is suppressed in HBV transgenic liver tumors of mouse models and in human HBV-related HCC. In HBV transgenic mice, heterozygous deletion of BECLIN 1 increases frequency of spontaneous malignancies and accelerated the onset of HCC (Qu et al., 2003); in line with this, the expression levels of *BECLIN 1* mRNA is lower in human HCC tissue than in chronic hepatitis tissue (Kotsafti et al., 2012). Lan et al. (2014) determined that autophagy selectively regulates the expression of the oncogenic miR-224 through autophagosome-mediated degradation. *In vitro* miR-224 plays its oncogenic role in hepatoma cell migration and tumor formation through silencing its target gene Smad4. The authors showed that autophagy is downregulated and inversely correlated with miR-224 expression in HBV-associated HCC patient specimens, and they demonstrated a significant correlation between low expression of ATG5, high miR-224, and low Smad4, with overall poor survival rate in HBV patients (Lan et al., 2014).

In conclusion, during infection, HBV upregulates and exploits the autophagy machinery for its replication and to establish a condition of chronic inflammation in the liver. However, when the disease gradually gets worse, autophagy is inhibited correlating with an increased progression of HBV-induced HCC. As autophagy could attenuate tumor progression through increasing autophagic cell death, antitumor immune response, and oncogenic microRNA degradation, it may represent a possible target for HCC treatment. To this regard, some studies have reported a positive effect of autophagy inducers on the survival of patients with HBV-related HCC (Cheng et al., 2009; Toso et al., 2010; Decaens et al., 2012).

### Hepatitis C Virus

Hepatitis C virus is an enveloped positive single-stranded RNA virus that belongs to the *Flaviviridae* family. HCV RNA encodes a single polyprotein precursor, which is cleaved by cellular and viral proteases into structural proteins, core, and the envelope glycoproteins E1 and E2, and non-structural (NS) proteins, p7 NS2, NS3, NS4A, NS4B, NS5A, and NS5B, which are involved in several processes essential for the completion of the HCV life cycle (Moradpour et al., 2007). The expression of HCV proteins induces extensive rearrangements of host cell membranes, thus leading to the formation of a complex compartment termed the membranous web, principally composed of double-membrane vesicles (DMVs), which, by harboring the viral replication machinery, allow viral RNA replication and assembly (Ferraris et al., 2013; Paul et al., 2013). Various viral proteins, such as Core, NS2, NS5B, NS3, and NS5A, were described to have a direct role in HCV-mediated carcinogenesis by inducing NF-KB activation, cell proliferation, genetic instability angiogenesis, or by inactivating apoptotic pathway, tumor suppressors, and immune response (Vescovo et al., 2016; Akram et al., 2017; Table 2). Besides the direct role of oncoviral proteins, HCV-mediated HCC progression has been proposed to be associated with alterations of the autophagic pathway during HCV infection (Figure 4).

Several studies have demonstrated that HCV induces autophagosome formation *in vitro* (Ait-Goughoulte et al., 2008; Sir et al., 2008; Dreux et al., 2009; Tanida et al., 2009), and an increased number of autophagosomes has been observed in liver biopsies obtained from patients chronically infected with HCV (Rautou et al., 2011). By exploiting the autophagic process, HCV promotes its own replication, interferes with the host immune response and lipid metabolism, thus favoring the establishment of chronic infection and HCV-associated pathogenesis (Vescovo et al., 2014).

Similar to HBV, HCV may also induce autophagy directly or indirectly, by promoting cellular stresses (e.g. ER and oxidative stress). In addition, it has been reported that HCV directly increases autophagosomes formation through the interaction of viral proteins with autophagic components. Specifically, P7 ion channel protein binds BECLIN 1 (Aweya et al., 2013), while NS5B has been described to associate with ATG5 and ATG12 (Guévin et al., 2010; Grégoire et al., 2011). ATG5 downregulation suppresses HCV RNA replication, supporting a role of autophagy and/or the interaction between ATG5 and NS5B in HCV RNA replication (Guévin et al., 2010), but is not clear yet whether the interaction between ATG5 and NS5B plays any role in the progression of autophagy in HCV-infected cells. In addition, viral NS3/4A have been found to interact with immunity-associated GTPase family M (IRGM), which associates with the autophagy-related proteins ATG5, LC3, BIF1, and ATG10 to induce autophagy (Grégoire et al., 2011). Furthermore, NS4B induces LC3 lipidation and forms complexes with Rab5, VPS34, and BECLIN (Grégoire et al., 2011). Furthermore, NS4B induces LC3 lipidation and forms complexes with Rab5, VPS34, and BECLIN 1 (Su et al., 2011), while the overexpression of NS5A upregulates the expression of BECLIN 1 (Shrivastava et al., 2012).

The induction of autophagy is essential for HCV RNA replication, and it has been proposed that autophagy plays a role in the biogenesis of viral replication compartments. Wang et al. (2017) have demonstrated that HCV-induced phagophores derive from the ER and serve as the assembly site for the HCV RNA replication complex, which remains associated to autophagosomes. Moreover, it has been reported that the autophagy elongation complex (ATG5-12/16L1) is recruited at the membranous web, where it promotes HCV replication, and ATG12 knockdown suppresses HCV RNA replication (Fahmy and Labonté, 2017). As autophagy activation could counteract HCV infection, the virus has evolved strategies to strengthen its persistency by temporally regulating the autophagic process. Indeed, HCV induces autophagy in the early stages of infection and simultaneously blocks the fusion between autophagosomes and lysosomes. Since autophagosomes act as platforms for the assembly of HCV RNA replication machinery, the virus accumulates autophagic vesicles and prevents their maturation to avoid the consequent degradation of viral replication complex into autolysosomes. The inhibition of autophagosome maturation is mediated by the HCV-related overexpression of Rubicon, which sequesters UVRAG from the homotypic fusion and protein-sorting complex. In the later stages of infection, the autophagic degradation is restored by the induction of UVRAG, which in turn antagonized the effect of Rubicon (Wang et al., 2015). The completion of autophagic flux, in the later stage of the HCV life cycle, allows HCV to degrade TRAF6 via autophagy. As TRAF6 is an adaptor molecule for the production of interferon and proinflammatory cytokines, HCV provides its autophagic degradation to suppress the host immune responses (Chan et al., 2016). In addition to sustaining HCV RNA replication, autophagy also regulates other important steps of the HCV life cycle. In particular, autophagy is necessary for the translation of incoming viral RNA, but it becomes dispensable once HCV replication is established (Dreux et al., 2009). Moreover, autophagy promotes viral transmission by enabling HCV release in the extracellular space, a mechanism which is impaired by the suppression of ATG7 and BECLIN 1 expression or by pharmacological inhibition of autophagy (Tanida et al., 2009; Shrivastava et al., 2011; Medvedev et al., 2017).

Although several studies have shown that HCV infection induces autophagy through the direct interaction of viral proteins with autophagy effectors, other numerous studies have shown that HCV induces autophagy by ER-stress, via induction of all three UPR pathways (PERK, ATF6, IRE1), and the inhibition of any of these three pathways decreases autophagy levels in HCV-infected cells (Ait-Goughoulte et al., 2008; Sir et al., 2008; Ke and Chen, 2011; Kim et al., 2013a; Chandra et al., 2014; Wang et al., 2014). Notably, the HCV core protein may induce autophagy through ER stress, by upregulating ATG12 and enhancing LC3B expression specifically through the activation of the PERK and ATF6 pathways (Wang et al., 2014). A relevant consequence of ER stress is calcium release, which impairs mitochondrial activity leading to the accumulation of ROS and damaged mitochondria. Similar to HBV, it has been shown that HCV promotes mitochondrial fission and mediates the removal of damaged mitochondria by inducing mitophagy, through the promotion of PINK1 and Parkin translocation on the outer surface of the mitochondria (Kim et al., 2013a). HCV-induced mitophagy correlates with deregulation of oxidative phosphorylation and depletion of mitochondria, which could contribute to liver injury (Kim et al., 2013a). Recently, it has been reported that the overexpression of NS5A is sufficient to induce Parkin translocation to the mitochondria and, consequently, mitophagy in a ROS-dependent manner (Jassey et al., 2019), while Core protein was demonstrated to inhibit mitophagy by inhibiting Parkin translocation to the mitochondria (Hara et al., 2014). A possible explanation to reconcile these conflicting evidences could be that, as well as bulk autophagy, HCV may also temporally regulate mitophagy depending on the expression level of NS5A and Core.

However, HCV usurps autophagy and mitophagy to reduce cellular stresses and to maintain cellular homeostasis. The removal of harmful components, such as damaged mitochondria, helps to promote cell survival by inhibiting apoptosis, resulting in HCV persistency and chronic infection. HCV induces autophagy and mitophagy to overcome ER and oxidative stress and to promote cell survival by inhibiting apoptosis, resulting in HCV persistency and chronic infection. By inducing ER stress, HCV also upregulates lipid biosynthesis, which represents a crucial step in HCV replication with severe consequence on pathogenesis, as lipid accumulation favors the development of hepatic steatosis. We have shown that autophagy in HCV-infected cells is also involved in preventing excessive accumulation of intracellular cholesterol, thus playing a protective role in the development of steatosis (Vescovo et al., 2012). In this regard, we recently demonstrated that inhibition of cholesterol synthesis by statins is not sufficient to prevent lipid accumulation when autophagy is inhibited, highlighting that deregulated lipoprotein uptake may also critically contribute to HCV-induced steatosis (Vescovo et al., 2019).

In addition, it has been characterized that HCV evades the host microbial defense responses by exploiting autophagy. Accordingly, HCV suppresses IFN signaling via autophagy, for example, by depleting TRAF6 through autophagic degradation, as mentioned above (Chan et al., 2016). Recently, NS5A was found to suppress both IFN signaling and autophagy by interacting with the mitochondrial autophagy inhibitor LRPPRC (Refolo et al., 2019). Conversely, knockdown of BECLIN 1, or ATG7 or ATG5, and treatment with autophagy inhibitors, such as chloroquine or Bafilomycin A, enhance the expression of type I interferon (IFN) and interferon-stimulated genes (ISGs) in HCV-infected cells (Ke and Chen, 2011; Shrivastava et al., 2011). In addition, HCV-induced autophagy is responsible for impaired expression of interferon-alpha receptor-1 (IFNAR1), which is essential for type I IFN signaling. Specifically, Kurt et al. have demonstrated that IFNAR1 is selectively degraded by chaperone-mediated autophagy (CMA) in HCV-infected cells (Kurt et al., 2015). CMA is a type of autophagy involved in selective lysosomal degradation of cytosolic proteins bearing a peculiar consensus motif (KFERQ). These proteins are recognized by the chaperone Hsc70, which translocates to the lysosome, where it binds to the CMA receptor LAMP2A (Kon and Cuervo, 2010).

Dash et al. (2019) have proposed that HCV-related induction of CMA has an essential role in hepatocarcinogenesis. In cirrhotic HCV-infected liver, they have reported that HCC progression is promoted by a switch from a protective state with high macro-autophagy and low chaperone-mediated autophagy (CMA) to a pro-cancer state characterized by low autophagy and high CMA (Dash et al., 2019). Supporting the idea that impairment in autophagy could promote HCC development, previous reports have shown that monoallelic deletion of BECLIN 1 results in an increased incidence of spontaneous HCC. In addition, loss of BECLIN 1 has been correlated with poor prognosis in various cancers, including HCC, in clinical studies (Qu et al., 2003; Shi et al., 2009; Qiu et al., 2014; Levine et al., 2015). Similarly, other studies have shown that mosaic deletion of ATG5 or hepatic disruption of ATG7 leads to spontaneous HCC development in mice (Takamura et al., 2011). Moreover, in HCC tissues of HCV-infected patients, an accumulation of p62 was observed, suggesting a block of the autophagic process, in the late stage of HCV pathogenesis. Specifically, p62 accumulation has been reported having an active role in carcinogenesis by activating the transcription factor Nrf2. Indeed, once phosphorylated, p62 is able to bind Keap1, thus impairing Keap1-mediated ubiquitination of Nrf2, which in turn translocates into the nucleus inducing cytoprotective genes, promoting cell survival and oncogenic signaling (Saito et al., 2016).

In addition to non-canonical p62/Nrf2 activation pathway, Nrf2 is strongly induced by ER and oxidative stress in persistently HCV-infected cells. Among Nrf2 target genes, Hsc70 and LAMP2 are upregulated in their transcription, consequently activating CMA (Aydin et al., 2018). The activation of CMA leads to lysosomal degradation of both pro-autophagic protein BECLIN 1, thus impairing autophagy, and the tumor suppressor p53 (Aydin et al., 2017, 2018). Accordingly, it has been shown that HCV replication is able to suppress p53 functions and that p53 protein levels are low in HCV-infected explanted livers (Aydin et al., 2017). Previous studies have reported that CMA compensate impaired autophagy in the cirrhotic liver to promote hepatocellular carcinoma (Chava et al., 2017). Therefore, altogether, the loss of p53, the impairment of autophagy, and the activation of CMA as pro-survival mechanism, could mediate the development of HCC in persistent HCV infection.

In summary, upon HCV infection, the autophagy process is strongly induced, as the virus needs autophagy to efficiently replicate, as well as to suppress host immune response. By exploiting autophagy, HCV could establish a condition of chronic inflammation in the liver. However, during chronic HCV infection, the constitutive activation of cellular stresses leads to autophagy inhibition and CMA activation, and both these events are involved in the development of HCV-related HCC.

## Lymphotropic Oncogenic Viruses

### Human Herpes Viruses

**FIGURE 5:**
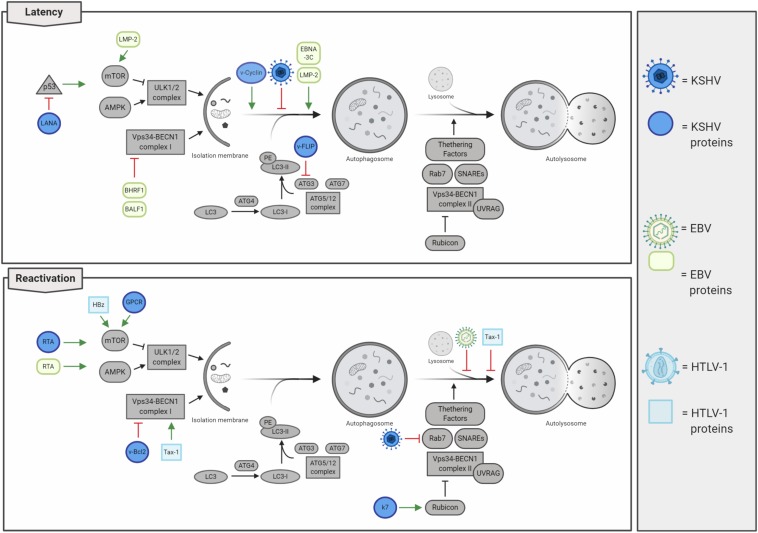
Lymphotropic oncogenic viruses and their impacts on autophagy regulation. Oncogenic herpesviruses [i.e. Kaposi’s sarcoma-associated herpesvirus (KSHV) and Epstein–Barr virus (EBV)] infect human cells rapidly establishing latency. Autophagy is mainly inhibited during latency, thus concurring in cancer development. In contrast, autophagy is promoted in lytic reactivation, even if the autophagosome maturation is inhibited. Human T-lymphotropic virus 1 (HTLV-1) negatively regulates autophagy by stimulating the activity of the mTOR kinase and by affecting autolysosome formation.

Human herpes viruses 4 and 8, also known as EBV and Kaposi’s sarcoma-associated herpesvirus (KSHV), respectively, belong to the sub-family of *Gammaherpesvirinae* with a large linear dsDNA surrounded by icosahedral capsid and an envelope. These viruses display a tropism for B lymphocytes, and both establish latent, as well as lytic infections promoting hematologic diseases and several solid human cancers (Chang et al., 2017). Interestingly, latent infections are mostly present in herpesvirus-associated malignancies, while the viral ability to straightly modulate autophagy depends on infective stages, changing between *de novo* infections, latency, or lytic reactivation (Cirone, 2018; Figure 5). Intriguingly, a common feature between lymphotropic oncoviruses resides in their ability to initially promote autophagy induction, which is followed by an inhibition of autophagosome maturation and a detrimental reduction in autophagy-mediated clearance, thus favoring tumorigenesis.

#### Kaposi’s Sarcoma-Associated Herpesvirus (KSHV)

Kaposi’s sarcoma-associated herpesvirus infection is associated with the development of several human pathologies, which include Kaposi’s sarcoma (KS), primary effusion lymphoma (PEL), multicentric Castleman’s disease (MCD), and inflammatory cytokine syndrome (KICS) (Cesarman et al., 2019). Besides B cells, KSVH infects monocytes, fibroblasts, endothelial, epithelial, and dendritic cells through the association with several cellular membrane receptors (integrin, heparan sulfate, xCT, ephrin A2, and DC-SIGN), and penetrates the host cell by viral glycoprotein-mediated membrane fusion (i.e. gB, gH, and gL) (Veettil et al., 2014). In *de novo* KSHV-infected cells, viral genome rapidly circularizes in the nucleus, where it is maintained in a latent state as a chromatinized plasmid; in these conditions, virions are not produced, and only a subset of genes are expressed (Yan et al., 2019). As a consequence of environmental changes and physiological stimuli (e.g. oxidative stress, hypoxia, and autophagy activation), KSHV-infected cells reactivate the viral cycle promoting the expression of lytic genes in a temporally regulated manner: immediate early, early, and late genes (Yan et al., 2019). Importantly, tumors associated with KSHV infection mostly present with viruses in a latent state; this observation led to the consideration of lytic reactivation not involved in tumorigenesis. Actually, it is recently emerging that both latency and lytic reactivation play a key role in KSHV-induced carcinogenesis (Aneja and Yuan, 2017). To this regard, it has been characterized that latent infection promotes proliferation and cell survival (Cesarman et al., 2019); by counterpart, virus reactivation not only ensures a higher dissemination of viral particles but also promotes angiogenesis (Asou et al., 1998; Wu et al., 2014) and the evasion of immunoresponse (Zhao et al., 2015), promoting cancer development. KSHV genome encodes several latent and lytic proteins that mimic cellular ones and usurp their functions, including those involved in autophagy regulation (Figure 5 and Table 2). During latency, autophagy is mainly inhibited. Although viral Cyclin D (vCyclinD) promotes autophagy induction by stimulating AMPK (Leidal et al., 2012), the viral oncoprotein FLIP (vFLIP) has been described to directly constrain autophagy by directly inhibiting ATG3 and LC3 activation (Lee et al., 2009). In addition both vCyclin D and vFLIP play a role in tumorigenesis; while the first interact with cellular CDK6 deregulating cell cycle, vFLIP suppresses apoptosis by inhibiting caspase 8 and NF-kB (Ganem, 2007). Another protein essential in latency and associated with autophagy inhibition is LANA, the latency-associated nuclear antigen. LANA is essential in maintaining viral genome in a chromatinized state and binds both viral and host DNA to avoid genome loss during mitosis, ensuring viral genome stability and replication (Yan et al., 2019). It has been described that LANA is a viral oncogene, which inhibits p53 activity protecting KSHV-infected cells from death and reduces pRB expression promoting cell proliferation (Friborg et al., 1999; Ganem, 2007). Considering that p53 mediates the transcription of several autophagic genes (White, 2016), the LANA-mediated p53 inhibition could contribute to autophagy impairment during KSHV latency. In addition, during latent infection, it has been reported that KSHV also constrains STAT3 (Roca Suarez et al., 2018), which in turn inhibits p53 and autophagy. Another relevant step during KSHV infections is represented by the lytic re-activation after latency, where autophagy stimulation is emerging to play a key role. To this regard, it has been shown that KSHV promotes STAT3 phosphorylation and activation, thus promoting autophagy and lytic re-activation (Santarelli et al., 2019). During this step, the viral Kaposin B and v-IL6 promote the phosphorylation of STAT3 on Ser727, promoting p53/p21 axis and autophagy inhibition (Deutsch et al., 2004; Suthaus et al., 2012; King, 2013; Santarelli et al., 2019). Moreover, it has been described that autophagy inhibition is able to reduce KSHV lytic reactivation (Wen et al., 2010). Wen et al. report that RTA, a viral protein essential in the re-activation of KSHV cycle, positively regulates autophagy induction. However, the lytic viral proteins GPCR and Bcl2 are able to inhibit autophagy through the activation of the PI3K/AKT/mTOR pathway and the alteration of BECLIN 1 activity and ATG14L stability (Pattingre et al., 2005; Bhatt and Damania, 2012; Zhang et al., 2015). These observations open to the possibility that, following an initial activation of autophagy during latent–lytic transition, autophagy is then inhibited to avoid viral degradation. Sustaining this hypothesis, it has been also described that the KSHV lytic protein K7 promotes the negative interaction between Rubicon and BECLIN 1 complex II, further blocking autophagosome maturation (Liang et al., 2013).

Altogether, these evidences highlight that autophagy is inhibited by KSHV during *de novo* infection and latency, activated to promote latency–lytic transition, and then rapidly blocked to complete the KSHV cycle (Cirone, 2018). To date, the extensive description on how KSHV regulates autophagy during viral life cycle needs to be implemented to elucidate whether the observed inhibition of autophagy, particularly during latency, directly contributes to oncogenesis.

#### Epstein–Barr Herpesvirus (EBV)

Similar to others, *Gammaherpesvirinae*, Epstein–Barr herpes virus (EBV), rapidly establishes latency as a consequence of infection in lymphoid tissues, promoting several lymphoproliferative disease. EBV can establish three different forms of latency: I, II, and III, which are characterized by the expression of precise subsets of EBV latent genes (Farrell, 2019). These different latent programs correlate with specific pathologies, e.g. the expression of all latent genes (latency III) is required for B-cell transformation and post-transplant lymphoma disorder (PTLD) development. On the other hand, Burkitt lymphoma (BL) is associated with latency I program and Hodgkin’s lymphoma (30% of cases), NK cell, and T-cell lymphomas, nasopharyngeal and gastric carcinomas with latency II (Farrell, 2019). Cellular proteins targeted by EBV and responsible for oncogenic transformation during latency have been extensively described (Farrell, 2019). Specifically, the latent oncogenic viral protein LMP-1 mimics a constitutively active CD40 receptor (CD40R), which promotes the NF-kB activation resulting in uncontrolled cellular proliferation and cell death inhibition (Mosialos et al., 1995; Kilger et al., 1998). In line with this, EBV latent membrane protein 2 (LMP-2) stimulates the PI3K/AKT pathway, a mechanism which inhibits autophagy and prevents apoptosis (Cen and Longnecker, 2015). LMP-2 has been also described to promote cell cycle progression by enhancing MYC-induced degradation of p27^kip1^, thus contributing to EBV-induced tumorigenesis (Fish et al., 2017). EBV proteins BHRF1 and BALF1 are orthologs of the cellular anti-apoptotic protein Bcl-2, which has been extensively described to negatively regulate both apoptosis and autophagy (Farrell, 2019). In addition, viral EBNA-1 is involved in carcinogenesis by acting on cellular environment and inducing genomic instability (Saridakis et al., 2005; Gruhne et al., 2009), EBNA-2 deregulates c-Myc expression increasing cell proliferation, and EBNA3A and EBNA3C repress both BIM and p16 and prevent differentiation into plasma cell (Akram et al., 2017; Farrell, 2019).

Interestingly, autophagy has been reported to be differently modulated not only between EBV latent and lytic cells but also among latency programs (Veettil et al., 2014). Dissimilar from other oncogenic viruses, it has been described that EBV preferentially promotes autophagy during latency II and III to maintain it and improve resistance to cell death, while no activation was described during the latency I program (Pujals et al., 2015). To this regard, it has been shown that the EBV latent protein LMP-1 is actively degraded through autophagy to properly maintain its expression levels, thus ensuring latency maintenance and preventing both cytostasis and immune recognition (Lee and Sugden, 2008). Moreover, EBV latent proteins LMP-2 and EBNA-3C promote autophagosome formation and upregulates the expression of pro-autophagic proteins (Fotheringham and Raab-Traub, 2015; Bhattacharjee et al., 2018). In contrast, autophagy is inhibited early at the induction step in *de novo* infections and later on at degradative phases during the viral reactivation from latency (Cirone, 2018). For example, the viral Bcl-2 orthologs BHRF1 and BALF1 have been described to inhibit BECLIN 1 and to be involved in mice chronic infection (Pattingre et al., 2005; Xiafoei et al., 2009); on the other hand, autophagy induction is promoted by the EBV lytic protein, Rta, via ERK1/2 activation and then suddenly blocked during the final phases of autophagy to avoid EBV virus elimination by lysosomal degradation (Granato et al., 2014; Hung et al., 2014). Moreover, autophagy directly contributes to MHC class II antigen presentation of viral EBNA1 peptides derived from autophagy degradation (Leung et al., 2010), a mechanism playing an essential role in antiviral immune response (Paludan et al., 2005). As a consequence of autophagy inhibition, it has been recently reported that the accumulation of p62 partially contributes to carcinogenesis by promoting genome instability in EBV-transformed B cells (Wang et al., 2019). In addition, it has been also described that Quercetin, a flavonoid acting as a potential anti-cancer molecule due to its anti-inflammatory and antioxidant properties, constrains both STAT3 activation and the autophagy inhibition promoted by EBV infection; this results in a reduction in IL-6, ROS, and p62, ultimately preventing B-cell immortalization (Granato et al., 2017, 2019). Overall, EBV infection leads to a reduction in autophagy clearance, rather than activation, thus, resulting in an accumulation of detrimental cell materials and promoting cancer development.

### Human T-Cell Lymphotropic Virus Type 1 (HTLV-1)

Human T-cell lymphotropic virus type 1 (HTLV-1) belongs to the genus *Deltaretrovirus* with a positive ssRNA genome encoding several genes, which include gag, pol, env, tax, and two long terminal repeats (Akram et al., 2017). HTLV-1 includes seven sub-types with a tropism for CD4+ T lymphocytes. It is estimated that HTLV-1 have infected approximately 10 million people worldwide, of which only about 2–5% progress to adult T-cell leukemia/lymphoma (ATLL), a very aggressive form of leukemia (Zhang et al., 2017). Different from animal retroviruses, where insertional mutations are responsible for carcinogenesis, it has been well characterized that HTLV-1 proteins, Tax-1 and HBz, are mainly responsible for ATLL development in humans (Akram et al., 2017). Specifically, HTLV-1 Tax-1 protein directly inhibits apoptosis and regulates the cell cycle by interacting with CREB (Yin et al., 1995). It promotes transcriptional activation through CBP/p300 (Kashanchi and Brady, 2005) and regulates several signaling pathways, e.g. IKK/NF-kB, DNA damage response, and innate immunity (Ishikawa and Barber, 2008).

Similarly to others lymphotropic oncogenic viruses, HTLV-1, on one hand promotes autophagy induction, on the other blocks autophagosomes maturation, thus resulting in obstructed process; this blockage could be even more detrimental for cells than the simply inhibition of induction steps, since accumulating autophagosomes could physically obstacle several cellular processes (Figure 4). In particular, Tax-1 has been described to stimulate the activity of the pro-autophagic protein BECLIN 1 by constantly activating the IKK complex and, in turn, NF-κB which promotes the formation of autophagosomes on lipid raft microdomains (Chen et al., 2015; Ren et al., 2015). In addition, Tax-1 inhibits the fusion between autophagosomes and lysosomes, finally resulting in the accumulation of autophagosomes (Tang et al., 2013). Another important protein involved in HTLV-1-induced tumorigenesis is represented by HBz, which endorses cell proliferation upregulating the expression of both brain-derived neurotropic factor (BDNF) and its receptor, TrkB (Polakowski et al., 2014). In addition, it has been reported that HBz inhibits both apoptosis and autophagy, strongly contributing to HTLV-1 mediated cancer development (Zhang et al., 2017). To this regard, Tanaka-Nakanishi et al. (2014) reported that HBz suppresses the pro-apoptotic protein Bim by negatively regulating its FoxO3a-mediated transcription. On the other hand, HBz constrains autophagy by negatively regulating the mTOR inhibitor, GADD34 (Mukai and Ohshima, 2014), which additionally results in cell growth and proliferation.

Overall, the reported findings highlight that autophagy is inhibited by HTLV-1, and this contributes to cancer development, together with cell death reduction and increased proliferation. However, further studies will be necessary to better define the temporal regulation of autophagy during HTLV-1 infection; this will not only lead to a more detailed understanding of ATLL development but could also inform on novel possible targets to both prevent and constrain HTLV-1-associated cancers.

## Conclusion

Virus-mediated oncogenesis has a multifaceted nature, which involved many cellular processes, such as cell cycle deregulation, inflammation, genetic instability, and autophagy. In addition, the alteration of other host factors independent of infection contributes to tumorigenesis, leading to the concept that oncogenic viruses promote cancer development, but the infection *per se* is not sufficient to induce carcinogenesis (Luo and Ou, 2015). Indeed, the incidence of viral-associated cancers is substantially lower than virus prevalence, and tumors occur in the context of persistent infections where the immune system is perturbed (e.g. immunosuppression or chronic inflammation) (Mesri et al., 2014). In the past decades, the molecular characterization of viral carcinogenesis led to the discovery of more than 30 cellular oncogenes and/or tumor suppressor genes, which were later found to be of enormous relevance in several forms of cancer (Chang et al., 2017). Importantly, viral oncoproteins strongly reprogram the assortment of the protein landscape in the host cells, acting on transcriptional regulation, as well as affecting proteome stability through the ubiquitin–proteasome system and autophagy (Akram et al., 2017). Autophagy is particularly targeted by viruses to avoid their elimination within the cell. To date, the known seven oncogenic viruses have been all described to impact on autophagy, with different outcomes depending on cellular tropism and infection stage (*de novo* chronic infection or viral cycle reactivation) (Silva and Jung, 2013). The genome of each oncogenic virus encodes specific oncogenes which affect autophagy, among other prominent cellular mechanisms. In particular, it is evident that autophagy is preferentially inhibited during *de novo* infection through a mechanism that can occur early on autophagy induction or late on autophagosome maturation, depending on the virus type, to avoid viral degradation (Mack and Munger, 2012). Latency is also mainly associated to autophagy inhibition (Cirone, 2018), although chronic infections could also promote selective autophagy induction as pro-survival response to virus-induced cellular stresses, thus ensuring viral persistency (e.g. HBV or HCV). In line with what was proposed for cancer development in general (Yun and Lee, 2018), it is emerging that autophagy inhibition contributes to tumorigenesis through a reduced efficiency of cell quality control in the course of infection with oncogenic viruses. In addition, tumor progression is often associated to a retrieval, whether or not an increase of autophagy representing an advantage for cancer cells, which overcomes both cellular and environmental stresses associated with cancer development. This is a more general mechanism that is independent from etiological factors concurring to cancer development (viral infection included) and is strongly connected to chemoresistance. To this regard, it is well established that autophagy inhibition could represent a valid strategy in cancer therapy, by reducing the resistance to treatments (Kögel, 2012; Levy et al., 2017). On the other hand, autophagy activation before tumor onset could promote both virus eradication and reduce cell damage, which are two main causes of tumor development. In light of the important contribution of autophagy alterations in virus-induced tumorigenesis, further studies will be necessary to deepen the molecular mechanisms by which each virus alters autophagy to identify more specific targets for new therapeutic approaches.

## Author Contributions

TV and MA conceptualized the work and prepared a draft of the manuscript text. BP contributed to figures development. MA and GF reviewed and edited the manuscript with suggestions from MP. All authors discussed the reported information and commented on the manuscript.

## Conflict of Interest

The authors declare that the research was conducted in the absence of any commercial or financial relationships that could be construed as a potential conflict of interest.
